# Neutrophils are not consistently activated by antineutrophil cytoplasmic antibodies in vitro

**DOI:** 10.1136/annrheumdis-2018-214405

**Published:** 2025-01-03

**Authors:** Reena J Popat, Michael G Robson

**Affiliations:** School of Immunology and Microbial Sciences, King's College London, London, UK

**Keywords:** systemic vasculitis, autoantibodies, inflammation

Antineutrophil cytoplasmic antibody (ANCA) vasculitis is characterised by autoantibodies against myeloperoxidase (MPO) and proteinase 3 (PR3). The evidence that ANCA are pathogenic comes from in vitro studies in which IgG from patients with anti-MPO or anti-PR3 antibodies activate neutrophils to undergo respiratory burst and degranulation. Furthermore, murine monoclonal antibodies against human MPO and PR3 and a chimeric humanised anti-PR3 monoclonal antibody activate neutrophils. The paradigm of neutrophil activation by ANCA has therefore become established.^1^ Further support for the pathogenicity of ANCA comes from in vivo studies in which injection of anti-MPO antibodies causes focal necrotising crescentic glomerulonephritis in mice.^2^

**Figure 1:**
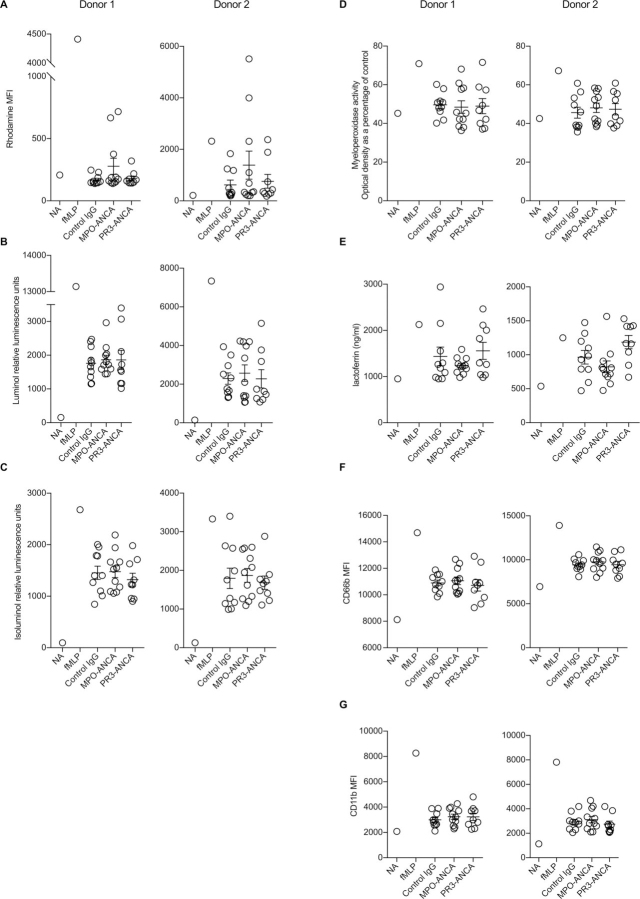
ANCA does not stimulate the neutrophil respiratory burst or degranulation in vitro. Ten control IgG, 11 MPO-ANCA and 9 PR3-ANCA were tested, with experiments performed in two neutrophil donors. The respiratory burst was assessed with (A) a dihydrorhodamine 123 assay of hydrogen peroxide generation, (B–C) luminol and isoluminol-based assays of total and extracellular superoxide generation. Degranulation products measured were (D) soluble MPO (azurophilic granules), (E) soluble lactoferrin (specific granules), (F) cell surface CD66b (specific granules) and (G) cell surface CD11b (secretory, gelatinase and specific granules). In (B–C), data shown are the peak response. For fMLP, this occurred at approximately 2 min, whereas the peak response to IgG was at approximately 30 min. There were no significant differences between the groups for any of the assays. ANCA, antineutrophil cytoplasmic antibody; fMLP, N-formylmethionine-leucyl-phenylalanine; NA, not activated.

We assessed the effect of purified ANCA on the activation of TNFα primed neutrophils using 10 control IgGs, 11 MPO-ANCA and 9 PR3-ANCA using two different assays of the neutrophil respiratory burst (full methods are in a online supplementary file 1). We found no significant difference in two separate neutrophil donors (figure 1A-C). We also used assays for four markers of neutrophil degranulation and found no differences in two neutrophil donors (figure 1D-G). The results are not due to inactivity of the purified ANCA IgG preparations. Aliquots of the same ANCA and control IgG batches were used in a recent publication where we demonstrated clear effects of these ANCA IgG preparations on monocytes, in experiments performed with during the same period of time.^3^

Our data challenge the established paradigm of neutrophil activation by ANCA. It is not clear why our results differ from others, but note that most previous publications have included small numbers which might lead to chance effects and selection bias. The ability of ANCA to activate neutrophils may be affected by affinity. We did not measure affinity or explore this possibility. We reviewed the literature to find publications in which six or more MPO-ANCA or PR3-ANCA IgG samples were compared with a similar number of control IgG samples and found only two. Franssen *et al* compared IgG purified from 17 PR3-ANCA positive patients, 14 MPO-ANCA positive patients and 16 controls. The patients were consecutive, eliminating selection bias.^4^ These authors found no significant effect of MPO-ANCA IgG on neutrophil respiratory burst using the DHR 123 and ferricytochrome C assays, and no effect on degranulation as measured by glucuronidase and lactoferrin release. There was an effect for PR3-ANCA which, although statistically significant, was small in magnitude. In all cases, the level of activation was much less than with N-formylmethionine-leucyl-phenylalanine. Harper *et al* compared 23 MPO-ANCAs, 15 PR3 ANCAs and 8 control IgGs using ferricytochrome C, calcium flux and MPO release assays.^5^ Both MPO-ANCA and PR3-ANCA caused significant activation compared with control IgG. However, in contrast to the study by Franssen *et al*, MPO-ANCA had a greater effect.

A recent report consistent with our data suggests that ANCA IgG does not activate neutrophils in vitro.^6^ Kraaij *et al* showed that serum from patients with ANCA vasculitis induced neutrophil extracellular traps (NET) formation, but this was unaffected by IgG depletion. In addition, purified IgG was unable to induce NET formation. This suggested that factors in the serum of patients with vasculitis, other than IgG, could activate neutrophils. This raises the possibility that the purity of IgG preparations could have influenced results in previous studies. We emphasise that our data do not exclude a role for neutrophils in the pathogenesis of ANCA vasculitis. ANCA may have direct or indirect effects on neutrophils in vivo that are not evident using in vitro assays of activation. We also acknowledge that there are many previous publications suggesting that ANCA do activate neutrophils in vitro and encourage other investigators to re-examine this question.
