# Supplementary material for: Neutrophils are not consistently activated by antineutrophil cytoplasmic antibodies in vitro
Source: Ann Rheum Dis. 2025 Jan 3;78(5):709–11. doi: 10.1136/annrheumdis-2018-214405 (PMC6517801; doi:10.1136/annrheumdis-2018-214405)
Supplement: Supplementary file 1 [file mmc1.pdf]

Full Methods

Purification of IgG from patients and controls

Blood or plasma exchange fluid was obtained from patients with ANCA vasculitis or healthy controls with ethical approval (NRES committee London—London Bridge 09/H084/72) and plasma stored at -80C. The clinical characteristics of the patients were included in the supplemental data of a previous publication (1). Samples were taken at presentation from patients with acute disease. Renal BVAS, total BVAS, other systems affected, and anti-MPO or PR3 levels, are reported. Fibrinogen was precipitated by adding 18g/100ml of sodium chloride and IgG was purified with protein G chromatography (GE Healthcare, Chalfont St Giles, UK). The endotoxin concentration in the final IgG preparations was measured by Lonza (Vervier, Netherlands) using a LAL kinetic chromogenic assay. For all 30 polyclonal IgGs used, the endotoxin was less than 0.05 eU/250µg IgG. IgG was stored at -20^{0}C and centrifuged at 16000g at 4^{0}C to remove aggregates before use. The concentration of IgG used for all neutrophil stimulation assays was 250µg/ml. We used an IgG concentration of 250 mcg/ml because it is the concentration used in the large majority of previous publications. However, the concentration in plasma is 5-10 mg/ml and it is possible that higher concentrations may have an effect *in vivo*.

Neutrophil Isolation

Heparinised blood from healthy controls was diluted 1:1 with Hanks Balanced Salt Solution (HBSS) and layer on Ficoll-Paque™ PLUS (GE Healthcare Life Sciences, UK) and centrifuged. After removing the upper layers, erythrocytes were lysed with ammonium chloride 0.83% (with 0.1mM EDTA and bicarbonate). The remaining neutrophils were washed in ice cold HBSS and resuspended in HBSS with 10mM hepes (HBH) or PBS with 0.33% sodium bicarbonate, 0.1% glucose, calcium and magnesium (PBS++). For all assays cells were primed by incubation with 2ng/ml TNFα for 15 minutes at 37^{0}C and subsequently incubated with IgG at 250 µg/ml at 37'C for one hour. fMLP at 100ng/ml was used as the positive control.

Dihydrorhodamine (DHR) 123 assay

Neutrophils were isolated as described above and used at a concentration of 2.5 x 10^6 cells/ml resuspended in HBH. The cells were loaded with 17µg/ml DHR123 (Calbiochem, UK) together with 5µg/ml Cytochalasin B and 2 mM sodium azide and incubated in the dark for 10 minutes at 37^0C. The reaction was stopped by the addition of a 30-fold volume of cold HBSS containing 1% BSA. This was spun at 280g and 4^0C for 5 minutes. The cells were resuspended in 300µls HBSS. Flow cytometry was performed on a FACSCanto flow cytometer (Becton Dickinson, UK) using FACSDIVA software (Becton Dickinson, UK). At least 10,000 events were collected per sample and data was analysed using FlowJo software (Treestar, USA). Data are expressed as median fluorescence intensity (MFI).

Luminometer assay

Neutrophils (final concentration 2.5 x10^6/ml were primed and stimulated at 37'C in a luminometer (Mithras LB940, Berthold Technologies, UK). A luminometer plate (PAA labs, UK) was coated with 200µls of FBS per well for 12 hours prior to use. Neutrophils were resuspended in PBS++. PBS++ with 50µ/ml HRP and 0.33mM luminol or isoluminol was prepared and mixed with the cell suspension in a ratio of 1.25:1. The stimulant (control IgG, ANCA, or fMLP) was dissolved in 20µl in the plate well, and 180µl of cells with luminol/isoluminol and HRP was added. Measurement started immediately.

Lactoferrin ELISA

Neutrophils were isolated as described at a concentration of 2.5x10^6 cells/ml resuspended in HBSS with 10mM Hepes. The cells were primed and stimulated as stated above. Supernatants were collected and stored at -20^0C. A human lactoferrin ELISA kit (Hycult Biotech, UK) was used according to the manufactuers' instructions. The plate was read using a SpectraMax Plus384 microplate reader (Molecular Devices, UK).

Myeloperoxidase detection

Neutrophils were isolated as described at a concentration of 2.5x10^6 cells/ml resuspended in HBSS with 10mM Hepes. The cells were primed and stimulated as stated above. Supernatants were collected and stored at -20^0C. 75µls/well of the supernatant was pipetted into respective ELISA plate wells and as a positive control 75µls of 2.5x10^6

neutrophils/ml was used. 100µls/well of OPD solution was added and incubated in the dark at room temperature for 30 minutes. The reaction was stopped by adding 100µls/well of 100% acetic acid. The plate was read at 450nm using a SpectraMax Plus384 microplate reader (Molecular Devices, UK).

CD66b and CD11b Flow cytometry

Neutrophils were isolated as described at a concentration of 2.5×10^6 cells/ml resuspended in HBSS with 10mM Hepes. The cells were primed and stimulated as stated above. Neutrophil activation was stopped by adding cold 1% BSA/HBSS and the cell suspension was then centrifuged at 380g for 5 minutes at 4^0C. The neutrophils were stained with anti-human antibodies CD66b FITC (clone G10F5, Becton Dickinson, UK) and CD11b APC (clone ICRF44, Becton Dickinson, UK). FACs analysis was carried out immediately after staining using a FACSCanto flow cytometer (Becton Dickinson, UK) and FACSDIVA software (Becton Dickinson, UK). At least 10,000 events were collected per sample and data was analysed using FlowJo software (Treestar, USA). Data are expressed as median fluorescence intensity (MFI).

1. Popat RJ, Hakki S, Thakker A, et al. Anti-myeloperoxidase antibodies attenuate the monocyte response to LPS and shape macrophage development,. *JCI Insight* 2017;2(2):e87379.
