# Supplementary material for: Neutrophils are not consistently activated by antineutrophil cytoplasmic antibodies in vitro
Source: Ann Rheum Dis. 2018 Dec 4;78(5):709–11. doi: 10.1136/annrheumdis-2018-214405 (PMC6517801; doi:10.1136/annrheumdis-2018-214405)
Supplement: Supplementary file 1 [file annrheumdis-2018-214405supp001.docx]

**Full Methods**

*Purification of IgG from patients and controls* 

Blood or plasma exchange fluid was obtained from patients with ANCA vasculitis or healthy controls with ethical approval (NRES committee London—London Bridge 09/H084/72) and plasma stored at -80C. The clinical characteristics of the patients were included in the supplemental data of a previous publication (1). Samples were taken at presentation from patients with acute disease.  Renal BVAS, total BVAS, other systems affected, and anti-MPO or PR3 levels, are reported. Fibrinogen was precipitated by adding 18g/100ml of sodium chloride and IgG was purified with protein G chromatography (GE Healthcare, Chalfont St Giles, UK). The endotoxin concentration in the final IgG preparations was measured by Lonza (Vervier, Netherlands) using a LAL kinetic chromogenic assay. For all 30 polyclonal IgGs used, the endotoxin was less than 0.05 eU/250µg IgG.  IgG was stored at -20^0^C and centrifuged at 16000g at 4^0^C to remove aggregates before use.  The concentration of IgG used for all neutrophil stimulation assays was 250µg/ml. We used an IgG concentration of 250 mcg/ml because it is the concentration used in the large majority of previous publications. However, the concentration in plasma is 5-10 mg/ml and it is possible that higher concentrations may have an effect *in vivo*.

*Neutrophil Isolation*

Heparinised blood from healthy controls was diluted 1:1 with Hanks Balanced Salt Solution (HBSS) and layer on Ficoll-Paque™ PLUS (GE Healthcare Life Sciences, UK) and centrifuged. After removing the upper layers, erythrocytes were lysed with ammonium chloride 0.83% (with 0.1mM EDTA and bicarbonate).  The remaining neutrophils were washed in ice cold HBSS and resuspended in HBSS with 10mM hepes (HBH) or PBS with 0.33% sodium bicarbonate, 0.1% glucose, calcium and magnesium (PBS++).  For all assays cells were primed by incubation with 2ng/ml TNFα for 15 minutes at 37^0^C and subsequently incubated with IgG at 250 µg/ml at 37’C for one hour.  fMLP at 100ng/ml was used as the positive control.

*Dihydrorhodamine (DHR) 123 assay*

Neutrophils were isolated as described above and used at a concentration of 2.5 x 10^6^ cells/ml resuspended in HBH. The cells were loaded with 17μg/ml DHR123 (Calbiochem, UK) together with 5μg/ml Cytochalasin B and 2 mM sodium azide and incubated in the dark for 10 minutes at 37^0^C. The reaction was stopped by the addition of a 30-fold volume of cold HBSS containing 1% BSA. This was spun at 280g and 4^0^C for 5 minutes. The cells were resuspended in 300μls HBSS. Flow cytometry was performed on a FACS Canto flow cytometer (Becton Dickinson, UK) using FACSDiva software (Becton Dickinson, UK). At least 10,000 events were collected per sample and data was analysed using FlowJo software (Treestar, USA).

*Luminometer assay*

Neutrophils (final concentration 2.5 x10^6^/ml were primed and stimulated at 37’C in a luminometer (Mithras LB940, Berthold Technologies, UK).  A luminometer plate (PAA labs, UK) was coated with 200μls of FBS per well for 12 hours prior to use.  Neutrophils were resuspended in PBS++.  PBS++ with 50μ/ml HRP and 0.33mM luminol or isoluminol was prepared and mixed with the cell suspension in a ratio of 1.25:1. The stimulant (control IgG, ANCA, or fMLP) was dissolved in 20µl in the plate well, and 180µl of cells with luminol/isoluminol and HRP was added. Measurement started immediately.

*Lactoferrin ELISA*

Neutrophils were isolated as described at a concentration of 2.5x10^6^ cells/ml resuspended in HBSS with 10mM Hepes.  The cells were primed and stimulated as stated above. Supernatants were collected and stored at -20^0^C.  A human lactoferrin ELISA kit (Hycult Biotech, UK) was used according to the manufactuers’ instructions. The plate was read using a SpectraMax Plus384 microplate reader (Molecular Devices, UK).

*Myeloperoxidase detection*

Neutrophils were isolated as described at a concentration of 2.5x10^6^ cells/ml resuspended in HBSS with 10mM Hepes. The cells were primed and stimulated as stated above. Supernatants were collected and stored at -20^0^C.  75µls/well of the supernatant was pipetted into respective ELISA plate wells and as a positive control 75µls of 2.5x10^6^ neutrophils/ml was used. 100µls/well of OPD solution was added and incubated in the dark at room temperature for 30 minutes. The reaction was stopped by adding 100µls/well of 100% acetic acid. The plate was read at 450nm using a SpectraMax Plus384 microplate reader (Molecular Devices, UK).

*CD66b and CD11b Flow cytometry*

Neutrophils were isolated as described at a concentration of 2.5x10^6^ cells/ml resuspended in HBSS with 10mM Hepes. The cells were primed and stimulated as stated above. Neutrophil activation was stopped by adding cold 1% BSA/HBSS and the cell suspension was then centrifuged at 380g for 5 minutes at 4^0^C. The neutrophils were stained with anti-human antibodies CD66b FITC (clone G10F5, Becton Dickinson, UK) and CD11b APC (clone ICRF44, Becton Dickinson, UK). FACs analysis was carried out immediately after staining using a FACS Canto flow cytometer (Becton Dickinson, UK) and FACsDIVA software (Becton Dickinson, UK).

1.	Popat RJ, Hakki S, Thakker A, et al. Anti-myeloperoxidase antibodies attenuate the monocyte response to LPS and shape macrophage development,. *JCI Insight* 2017;2(2):e87379.
